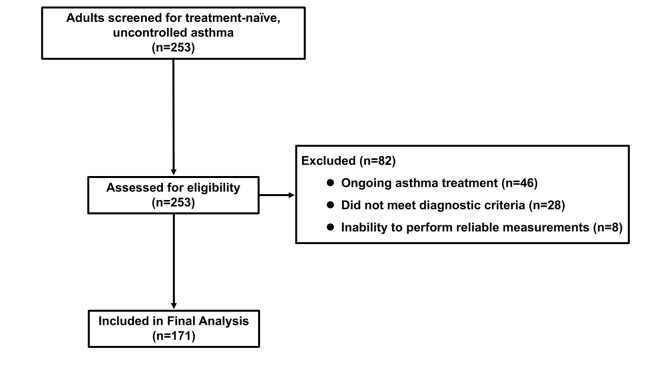# Phenotyping treatment-naive uncontrolled asthma in adults: A primary care framework

**DOI:** 10.1016/j.jacig.2026.100690

**Published:** 2026-03-20

**Authors:** Yutaka Nakano, Rika Nakano, Takuya Yukawa, Akihiro Arita

**Affiliations:** a Nakano Respiratory and Allergy Clinic, Hamamatsu, Japan; b AOI Pharmacy, Hatsuoiten, Japan

**Keywords:** Asthma, phenotype, treatment-naive, primary care, small airway dysfunction, type 2 inflammation, treatable traits, cluster analysis, biomarkers, personalized medicine

## Abstract

**Background:**

Initial asthma management often relies on a one-size-fits-all approach; however, the heterogeneity of treatment-naive, uncontrolled asthma remains insufficiently characterized. Specifically, the complex relationships among symptom burden, type 2 (T2) inflammation, and pulmonary function in this population are poorly understood.

**Objective:**

We sought to identify distinct clinical phenotypes in treatment-naive adults via multidimensional analysis and evaluate the association between symptom scores and T2 inflammation.

**Methods:**

This single-center, retrospective study analyzed 171 treatment-naive adults with uncontrolled asthma (5-item Asthma Control Questionnaire [ACQ-5] score ≥ 1.5). We performed k-means clustering using 20 variables, including demographics, symptoms, T2 biomarkers (blood eosinophils and fractional exhaled nitric oxide), and lung function (FEV_1_ and forced expiratory flow at 25% to 75% of forced vital capacity). Logistic regression assessed the ability of ACQ-5 scores to predict T2-high status (blood eosinophils ≥ 300/μL and fractional exhaled nitric oxide > 50 ppb).

**Results:**

Cluster analysis identified 3 phenotypes: relative T2-low/preserved function (32.7%); severe T2/obstructive (29.2%); and (3) T2-moderate/small airway dysfunction predominant (38.0%). The prevalence of T2-high status varied significantly (30.4%, 64.0%, and 50.8%, respectively; *P* = .002). ACQ-5 scores showed limited ability to discriminate T2-high status (area under the curve 0.615). Multivariable modeling provided only modest improvement (area under the curve 0.644).

**Conclusions:**

In this study population, treatment-naive uncontrolled asthma comprises 3 clinically distinct phenotypes with divergent inflammatory and functional profiles. Symptom burden alone appeared to be a limited predictor of T2-high inflammation. These exploratory findings suggest that incorporating objective biomarkers and functional assessments into initial care may help tailor therapeutic strategies for patients with small airway dysfunction or discordant symptom-inflammation profiles. Further validation in larger cohorts is required.

## Graphical abstract

**Figure:**
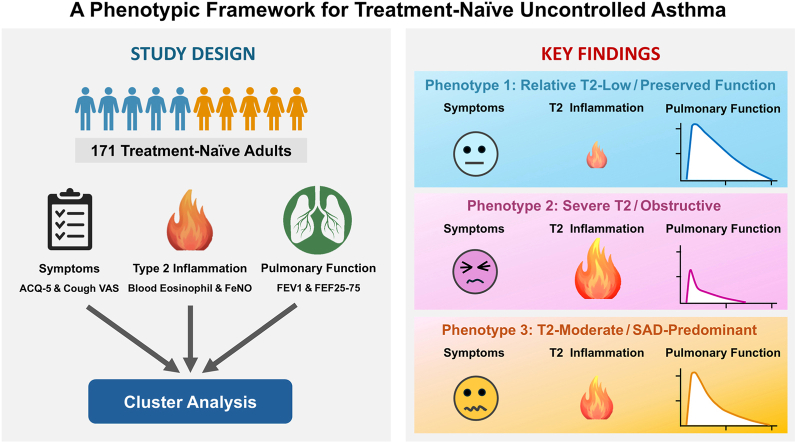

Asthma affects more than 300 million individuals worldwide; however, therapeutic failures persist despite substantial advances in treatment.^1^ Real-world evidence reveals that in 35% to 46% of patients in the United Kingdom who initiate medium- or high-dose inhaled corticosteroid (ICS)/long-acting β_2_-agonist (LABA) therapy, asthma remains uncontrolled,^2^ whereas more than 20% of Japanese patients experience inadequate control after treatment initiation.^3^ These findings underscore persistent limitations in current management strategies.

International guidelines, including the 2024 Global Initiative for Asthma (GINA) recommendations, advocate stepwise treatment escalation primarily on the basis of symptom control.^4^^,^^5^ However, this one-size-fits-all strategy does not adequately address the multidimensional nature of asthma, which encompasses symptom burden, type 2 (T2) inflammation, and airflow limitation—domains that are often discordant.^6^ This oversimplification likely contributes to suboptimal outcomes and underscores the imperative for more individualized approaches to initial therapy.^7^

The precision medicine paradigm, particularly the treatable traits framework, has transformed the management of severe, treatment-refractory asthma by emphasizing interventions tailored to underlying pathophysiologic mechanisms.^8^^,^^9^ Although this approach has resulted in important progress in advanced disease,^10^, ^11^, ^12^, ^13^ treatment-naive asthma remains insufficiently studied. In particular, the interplay among symptoms, inflammatory status, and pulmonary function, including often underrecognized features such as small airway dysfunction (SAD), in shaping clinical phenotypes at the time of initial therapeutic decision making is largely unexplored.^14^, ^15^, ^16^, ^17^, ^18^ Characterizing this natural heterogeneity, unconfounded by prior treatment, is essential for optimizing precision-based initial management.

This issue is especially relevant in primary care, where most asthma care is initiated, but access to comprehensive biomarker testing is limited.^19^, ^20^, ^21^ As a result, therapeutic decisions frequently rely on symptom-based instruments such as the 5-item Asthma Control Questionnaire (ACQ-5).^22^^,^^23^ However, the extent to which such tools reflect T2 inflammatory activity remains uncertain. Accordingly, this study aimed to identify distinct clinical phenotypes in treatment-naive adults with uncontrolled asthma through unsupervised cluster analysis of integrated symptom, inflammatory, and pulmonary function profiles. We further evaluated the utility of ACQ-5 as a surrogate marker of T2 inflammation and sought to develop a conceptual framework for phenotype-guided initial therapy.

## Methods

### Study design and participant selection

This single-center, retrospective, cross-sectional study evaluated consecutive treatment-naive adults with uncontrolled asthma who attended a primary care clinic specializing in respiratory and allergic disorders in Japan between April 2020 and March 2022. The study protocol was approved by the local Institutional Review Board (approval no. 22-02, dated August 30, 2022) and was conducted in accordance with Good Clinical Practice guidelines and Declaration of Helsinki principles. Because of the retrospective design, informed consent was waived, and all data were anonymized. Participants were informed of the study through clinic notices and institutional websites, with explicit opt-out options.

### Inclusion and exclusion criteria

Eligible participants were adults (≥ 18 years old) with a physician-confirmed diagnosis of asthma according to GINA criteria. All baseline assessments for this study were conducted at the initial visit before the initiation of any controller therapy. Eligibility required meeting 1 of the following criteria: (1) documented history of characteristic respiratory symptoms (wheeze, dyspnea, chest tightness, and cough with temporal and intensity variability) or (2) for patients whose symptoms were less typical, objective evidence of reversible expiratory airflow limitation, confirmed by an improvement in FEV_1_ ≥ 12% and ≥ 200 mL from baseline following a minimum 4-week course of ICS-containing therapy initiated as part of routine clinical care. This dual-criteria diagnostic approach, grounded in the GINA strategy, was implemented to rigorously define the asthma cohort and minimize the potential inclusion of overlapping conditions, particularly cough-variant asthma.

Uncontrolled asthma was defined as an ACQ-5 score ≥ 1.5.^22^ Treatment-naive status was defined as the absence of any asthma controller medications, including ICS, LABA, long-acting muscarinic antagonists (LAMA), inhaled combination therapies, oral corticosteroids, leukotriene receptor antagonists, theophylline, and biologics, for at least 6 months before the baseline visit.

The key exclusion criteria were as follows: (1) pneumonia within the preceding 8 weeks; (2) significant cardiorespiratory comorbidities (eg, congestive heart failure, bronchiectasis); (3) a smoking history of ≥ 10 pack-years coupled with symptom onset after age 40 (this criterion was applied to minimize the inclusion of patients with a high probability of chronic obstructive pulmonary disease or asthma and chronic obstructive pulmonary disease overlap^5^; current smokers who did not meet this specific exclusion criterion remained eligible for inclusion); (4) use of short-acting β_2_-agonists within 6 hours before the clinic visit; (5) inability to perform reliable spirometry or fractional exhaled nitric oxide (Feno) measurements according to quality standards; (6) current pregnancy or lactation.

### Data collection and measurements

At baseline, demographic data, smoking status, and comorbidities including allergic rhinitis and chronic sinusitis were recorded. Symptom severity was assessed using the validated Japanese version of the ACQ-5.^22^ Cough severity was measured using a 100-mm visual analog scale.

Pulmonary function was assessed using standardized spirometry (P-370; Fukuda Denshi, Tokyo, Japan) following American Thoracic Society/European Respiratory Society recommendations.^24^ At least 3 acceptable forced expiratory maneuvers were obtained, with the best values recorded: forced vital capacity (FVC), FEV_1_, and forced expiratory flow at 25% to 75% of the FVC (FEF_25-75_).^25^ Results were expressed as absolute values and percentages of predicted values, calculated using established Japanese reference equations.^26^

T2 inflammatory biomarkers included peripheral blood eosinophil counts (XN-9100; Sysmex, Kobe, Japan), Feno (NIOX VERO; Circassia AB, Uppsala, Sweden), and total serum IgE (Phadia 2500; Thermo Fisher Scientific, Waltham, Mass). T2-high status was defined as concurrent blood eosinophil counts ≥ 300 cells/μL and Feno > 50 ppb, based on evidence supporting their predictive utility. Post hoc analyses of the CAPTAIN trial demonstrated that patients with this biomarker profile derived greater benefit from high- versus medium-dose ICS-containing therapy.^27^^,^^28^

### Statistical analysis

Analyses were conducted using R version 4.5.0 (https://www.R-project.org/) with EZR.^29^ Continuous variables were expressed as median and interquartile range, and categorical variables were expressed as frequencies and percentages.

### Unsupervised clustering and phenotype identification

To identify clinical phenotypes, k-means clustering was applied to 171 patients with complete data across 20 variables selected for their relevance to asthma pathophysiology and the treatable traits framework.^14^, ^15^, ^16^ These included demographic factors (sex, age, body mass index, smoking status, allergic rhinitis, and chronic sinusitis), inflammatory markers (blood eosinophil and neutrophil counts, log-transformed total serum IgE and Feno), lung function (FVC percentage of predicted value, FEV_1_ percentage of predicted value, FEF_25-75_ percentage of predicted value), and symptom measures (ACQ-5 total and subdomain scores and cough visual analog scale score). Continuous variables were standardized to *z* scores, and categorical variables were converted into dummy variables.

The optimal cluster number was assessed using multiple validation metrics, including silhouette scores.^30^ Average silhouette values were modest (k = 2:0.173; k = 3:0.109; k = 4:0.111), consistent with a continuous clinical spectrum. Although k = 2 was statistically favored, it produced an overly simplistic dichotomy based mainly on T2 inflammation. The k = 4 model generated an unstable cluster with substantial overlap. Therefore, we selected the k = 3 solution, as it provided the most clinically meaningful and interpretable differentiation. Specifically, the k = 3 model uniquely isolated a large, therapeutically relevant T2-moderate/SAD-predominant phenotype that would have been otherwise missed. This selection prioritized clinical interpretability over purely statistical criteria, aligning with best practices in clinical phenotyping.^31^ The k-means algorithm used 25 random initializations to ensure stability.

### Intergroup comparisons and predictive modeling

Phenotypic differences were assessed using the Kruskal-Wallis test for continuous variables and the χ^2^ test for categorical variables. Significant results were followed by post hoc pairwise analyses: Dunn’s test with Bonferroni correction for continuous data and pairwise χ^2^ tests with Bonferroni adjustment for categorical data.

To identify predictors of T2-high status, logistic regression was performed. Variables with a *P* value of <.20 in univariate analysis, together with the ACQ-5 score (the primary predictor of interest), were entered into a multivariate model. Odds ratios with 95% CIs were calculated. Model performance, and that of ACQ-5 alone, was evaluated using the area under the curve (AUC). A 2-sided *P* value of <.05 was considered statistically significant.

## Results

### Patient selection and baseline characteristics

Of 253 adults screened, 171 (67.6%) met the eligibility criteria and were included in the final analysis. The main reasons for exclusion were prior or ongoing asthma treatment (n = 46), failure to meet the diagnostic criteria after a 4-week trial of ICS-containing therapy (n = 28), and inability to perform reliable spirometry or Feno measurements (n = 8) (Fig E1 in this article’s Online Repository available at www.jaci-global.org).

The baseline characteristics of the study population are summarized in Table I. The median age of the cohort was 46 years, and sex was evenly distributed (50.3% women). Adult-onset asthma was predominant (67.8%). By definition, the participants had poorly controlled asthma, with a median ACQ-5 score of 3.0 (interquartile range, 2.4-3.6). Most participants (91.2%) were deemed to require GINA step 4 or 5 therapy at initiation.

**Table I:** Baseline demographics and clinical characteristics of patients (N = 171)

Characteristics	Values
Age (y), median (IQR)	46 (36-59)
Sex, female, no. (%)	85 (49.7)
Age of asthma onset, no. (%)	
Childhood	55 (32.2)
Adult	116 (67.8)
BMI (kg/m^2^), median (IQR)	23.0 (20.8-25.6)
Current smoker, no. (%)	33 (19.3)
Comorbidities, no. (%)	
Allergic rhinitis	134 (78.3)
Sinusitis	46 (26.9)
ACQ-5 score, median (IQR)	3.0 (2.4-3.6)
Lung function, median (IQR)	
FVC (L)	3.15 (2.67-4.07)
FVC% predicted (%)	95.1 (84.3-103.1)
FEV_1_ (L)	2.47 (1.93-3.15)
FEV_1_% predicted (%)	86.8 (75.0-96.8)
FEV_1_/FVC (%)	77.0 (69.6-83.1)
FEF_25-75_ (L/s)	2.18 (1.52-2.94)
FEF_25-75_% predicted (%)	60.1 (41.0-79.5)
Inflammatory biomarkers, median (IQR)	
Blood eosinophils (cells/μL)	374 (217-612)
Feno (ppb)	69 (36-111)
Total serum IgE (IU/mL)	239 (91-753)
Log total serum IgE	2.4 (2.0-2.8)

*BMI*, Body mass index; *IQR*, interquartile range; *% predicted*, percentage of predicted value.

### Identification of 3 distinct clinical phenotypes

**Fig 1:**
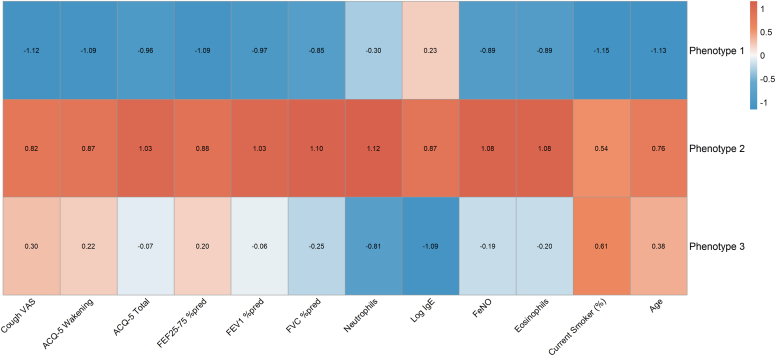
Heatmap of standardized clinical variables across 3 phenotypes. Heatmap visualizes the mean standardized values (*z* scores) of the key clinical variables for each of the 3 identified phenotypes. Each column represents a clinical variable, and each row represents a phenotype. The color scale indicates the relative value of each variable within that column, with *red-brown* tones representing higher values (eg, more severe symptoms, higher inflammation) and *blue* tones representing lower values (eg, better lung function, lower inflammation) compared with the mean of the entire cohort for that variable. The numeric value within each cell is the *z* score. *%pred*, Percentage of predicted value; *VAS*, visual analog scale.

**Fig 2:**
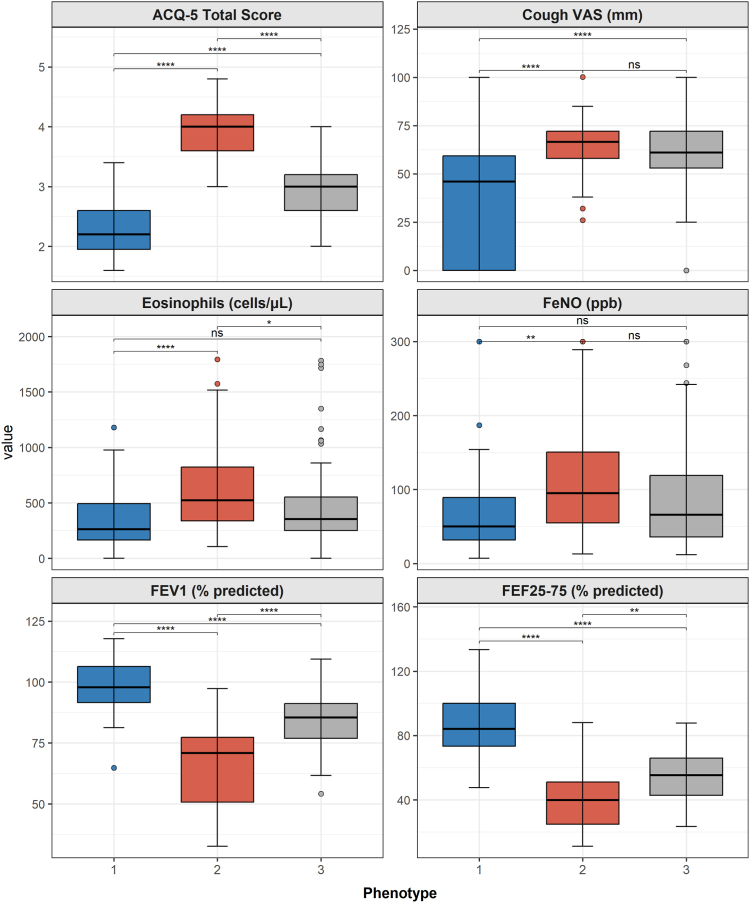
Boxplot comparisons of key clinical variables across 3 phenotypes. Boxplots compare the distribution of 6 key variables across phenotype 1 (relative T2-low/preserved function), phenotype 2 (severe T2/obstructive), and phenotype 3 (T2-moderate/SAD predominant). The *central line* in each box represents the median, the *box edges* represent the interquartile range, and the whiskers extend to 1.5 × interquartile range. The *circles* represent the outliers. Statistical comparisons were made using the Kruskal-Wallis test, followed by post hoc Dunn’s tests. ∗*P* < .05; ∗∗*P* < .01; ∗∗∗*P* < .005; ∗∗∗∗*P* < .001. *ns*, Not significant; *% predicted*, percentage of predicted value; *VAS*, visual analog scale.

Unsupervised k-means clustering of the 20 variables identified 3 distinct and clinically interpretable phenotypes (Table II and Fig 1). These were designated according to their dominant features: (1) relative T2-low/preserved function, (2) severe T2/obstructive, and (3) T2-moderate/SAD predominant. Significant differences were observed across the inflammatory, functional, and symptom domains (Fig 2).

**Table II:** Comparison of clinical and demographic characteristics across 3 phenotypes

Variables	Phenotype 1 (n = 56)	Phenotype 2 (n = 50)	Phenotype 3 (n = 65)	*P* value
Demographics				
Age (y), median (IQR)	43 (33-54)	51 (37-60)	49 (40-61)	.072 (ns)
BMI (kg/m^2^), median (IQR)	22.4 (21.1-25.3)	22.8 (21.1-25.9)	23.4 (20.5-25.0)	.964 (ns)
Comorbidities, no. (%)				
Sinusitis	16 (28.6)	11 (22.0)	19 (29.2)	.653 (ns)
Allergic rhinitis	44 (78.6)	40 (80.0)	50 (76.9)	.970 (ns)
Smoking status, no. (%)				.179 (ns)
Current smoker	5 (8.9)	12 (24.0)	16 (24.6)	
Ex-smoker	19 (33.9)	14 (28.0)	21 (32.3)	
Never-smoker	32 (57.1)	24 (48.0)	28 (43.1)	
Laboratory findings, median (IQR)				
Eosinophils (cells/μL)	262 (166-492)^b^	523 (339-823)^a^	353 (250-553)^b^	< .001
Feno (ppb)	50 (32-89)^b^	95 (55-151)^a^	66 (36-119)^ab^	< .01
Type 2 high (%)	17 (30.4)^b^	32 (64.0)^a^	33 (50.8)^a^	< .01
Log IgE	2.43 (2.04-2.76)^ab^	2.55 (2.12-2.87)^a^	2.17 (1.70-2.64)^b^	< .05
Neutrophils (cells/μL)	3669 (2843-4573)	4183 (3287-5146)	3482 (2720-4651)	.061 (ns)
Pulmonary function, median (IQR)				
FVC% predicted	100.6 (95.6-107.6)^a^	82.1 (68.6-93.0)^c^	94.9 (88.5-107.5)^b^	< .001
FEV_1_% predicted	97.9 (91.6-106.4)^a^	70.9 (50.7-77.4)^c^	85.5 (76.9-91.2)^b^	< .001
FEF_25-75_% predicted	84.2 (73.4-100.1)^a^	40.0 (24.8-51.2)^c^	55.3 (42.9-65.9)^b^	< .001
ACQ-5 scores, median (IQR)				
Nocturnal wakening	3 (2.0-3.0)^c^	6 (5.0-6.0)^a^	5 (4.0-5.0)^b^	< .001
Morning symptoms	3 (2.8-4.0)^c^	5 (4.0-5.0)^a^	4 (4.0-5.0)^b^	< .001
Activity limitation	2 (2.0-3.0)^b^	3 (3.0-4.0)^a^	3 (2.0-3.0)^a^	< .001
Dyspnea	2 (1.0-2.0)^b^	3 (3.0-3.0)^a^	2 (1.0-3.0)^b^	< .001
Wheeze	2 (0.0-2.0)^b^	4 (3.0-4.0)^a^	2 (0.0-3.0)^b^	< .001
Total score	2.2 (2.0-2.6)^c^	4.0 (3.6-4.2)^a^	3.0 (2.6-3.2)^b^	< .001
Cough VAS	46 (0-59)^b^	67 (58-72)^a^	61 (53-72)^a^	< .001

Overall *P* values for comparisons across the 3 phenotypes were derived from Kruskal-Wallis test for continuous variables and χ^2^ test (or Fisher’s exact test where appropriate, eg, if expected cell counts were < 5) for categorical variables. If the overall test was significant (*P* < .05), pairwise comparisons for continuous variables were performed using Dunn’s post hoc test with Bonferroni correction, and pairwise comparisons for categorical variables (specifically for T2-high status in this table) were performed using χ^2^ tests with Bonferroni correction on 2 × 2 contingency tables. Values in the same row that do not share a common superscript letter (a, b, c) are significantly different (*P* < .05) based on these post hoc tests.

*BMI*, Body mass index; *IQR*, interquartile range; *ns*, not significant (*P* ≥ .05); *% predicted*, percentage of predicted value; *VAS*, visual analog scale.

#### Phenotype 1: Relative T2-low/preserved function (n = 56, 32.7%)

This group exhibited a notable symptom burden (median ACQ-5 score 2.2) despite preserved lung function and the lowest T2 inflammation. The median FEV_1_ (97.9% predicted) and FEF_25-75_ (84.2% predicted) were significantly higher, whereas blood eosinophils (262 cells/μL) and Feno (50 ppb) were significantly lower than values observed in the more inflamed groups (*P* < .01 for key comparisons).

#### Phenotype 2: Severe T2/obstructive (n = 50, 29.2%)

This group demonstrated the most severe disease profile, characterized by high T2 inflammation, marked airflow limitation, and greatest symptom burden. The median blood eosinophil count (523 cells/μL) and Feno (95 ppb) were the highest among the groups. Lung function was most impaired, with the lowest median FEV_1_ (70.9% predicted) and FEF_25-75_ (40.0% predicted) (*P* < .001 vs others). The symptom burden was also the greatest, with a median ACQ-5 score of 4.0.

#### Phenotype 3: T2-moderate/SAD predominant (n = 65, 38.0%)

The largest subgroup was characterized by intermediate T2 inflammation and a functional impairment pattern suggestive of SAD. The median FEV_1_ was moderately reduced (85.5% predicted), whereas the median FEF_25-75_ was disproportionately low (55.3% predicted), positioned between the preserved function and obstructive groups. The symptom burden and inflammatory markers were also intermediate.

### Distribution of T2-high status across phenotypes

The prevalence of T2-high status (eosinophils ≥ 300/μL and Feno > 50 ppb) differed significantly among the phenotypes (*P* = .002). It was highest in the severe T2/obstructive group (64.0%), intermediate in the T2-moderate/SAD predominant group (50.8%), and lowest in the relative T2-low/preserved function group (30.4%). The prevalence of the severe T2/obstructive phenotype was significantly higher than that of the relative T2-low/preserved function group (*P* = .003).

### Symptom severity as a predictor of T2-high status

Next, we evaluated whether symptom severity predicted T2-high inflammation. In univariate logistic regression, higher ACQ-5 scores were associated with T2-high status (odds ratio = 1.62 per 1-point increase; 95% CI, 1.11-2.40; *P* = .014). However, its discriminatory performance was poor, with an AUC of 0.615. An optimal ACQ-5 score cutoff of 3.1 provided limited sensitivity (57.3%) and specificity (67.4%).

Adding other clinical variables to the multivariate model did not substantially improve its performance (AUC 0.644). In this model, neither the ACQ-5 score (*P* = .057) nor comorbid chronic sinusitis (*P* = .057) remained statistically significant, showing only a trend. Taken together, these results indicate that symptom-based tools are unreliable surrogates for identifying T2-high inflammation in this population.

## Discussion

### A new framework for an old problem: deconstructing treatment-naive asthma

This study presents a multidimensional analysis of treatment-naive adults with uncontrolled asthma in a primary care setting. Our exploratory cluster analysis suggests that this population is not homogeneous and may comprise at least 3 clinical phenotypes, each characterized by a distinct interplay of symptom burden, T2 inflammation, and pulmonary function. These findings highlight the complexity of the disease, even in the treatment-naive state, and suggest that a uniform therapeutic strategy may not adequately address the specific pathophysiology of all patients.

### Situating the phenotypic spectrum within the existing landscape

Two of the identified phenotypes align with established asthma constructs. The severe T2/obstructive phenotype represents the archetypal high-risk patient, characterized by a triad of severe symptoms, profound airflow limitation, and florid T2 inflammation. This distinct profile renders this group readily identifiable and implies a need for prioritized, intensive management. Conversely, the relative T2-low/preserved function phenotype, with its lower (though still significant) symptom burden, relatively preserved lung function, and comparatively lower levels of T2 inflammation, mirrors the patient population for whom conventional, symptom-guided treatment escalation was originally developed.^4^^,^^5^

However, the most prevalent cluster, the T2-moderate/SAD predominant phenotype, occupies a crucial and often overlooked space between these 2 poles. Characterized by moderate inflammation and a disproportionate reduction in FEF_25-75_, this group highlights the significant role of SAD in individuals with untreated asthma. Although SAD is a well-documented feature of severe, refractory asthma,^17^^,^^18^ the characteristics of this group highlight its potential role in untreated asthmatics. This does not establish a new endotype,^32^ but rather suggests that SAD may be a foundational element in the natural history of uncontrolled asthma, even before therapeutic interventions.

### Reframing initial treatment strategies: A phenotype-driven approach

Our phenotypic framework offers a conceptual blueprint for tailoring initial asthma therapy to the underlying pathophysiology. Although prospective validation is essential, these considerations form a rational basis for future clinical trials.

### Phenotype 1 (relative T2-low/preserved function): The guideline-concordant phenotype

This phenotype represents a common clinical presentation: patients with a significant symptom burden requiring intervention, but with relatively preserved lung function and a T2 inflammatory load that, though present, is the lowest among the 3 groups. Their profile aligns well with the patient populations for whom standard GINA-recommended therapies were primarily developed. Consequently, initiating current guideline-concordant therapy, such as low- to medium-dose ICS/formoterol maintenance and reliever therapy (preferred) or fixed-dose ICS/LABA with as-needed short-acting β_2_-agonists (alternative), appears to be a rational and evidence-based starting point.^4^^,^^5^ This group serves as a crucial reference, highlighting the distinct pathophysiologic features of the other 2 phenotypes that may require a more tailored approach.

### Phenotype 2 (severe T2/obstructive): An argument for early, aggressive intervention

In stark contrast, this phenotype represents a high-risk profile where severe symptoms, intense T2 inflammation, and profound airflow limitation converge. These patients are likely predisposed to frequent exacerbations and early treatment failure under standard step-up care.^2^^,^^3^ Consequently, a more intensive upfront strategy aimed at rapidly gaining control and potentially altering this high-risk trajectory may be warranted. The marked T2-high status predicts robust corticosteroid responsiveness, justifying consideration of a brief initial oral corticosteroid course for stabilization.^33^ Evidence from the CAPTAIN trial showing superior outcomes in patients with T2-high status with high-dose ICS supports this approach.^27^^,^^28^ Furthermore, given the severe airway impairment, the addition of a LAMA to form single-inhaler triple therapy represents a rational option to maximize bronchodilation and gain rapid control,^34^^,^^35^ especially as high-dose maintenance and reliever therapy is not recommended by GINA. This intensive approach necessitates a clear plan for systematic de-escalation once control is achieved to minimize long-term corticosteroid-related adverse effects.^36^^,^^37^

### Phenotype 3 (T2-moderate/SAD predominant): Potential relevance of small airway dysfunction

The identification of the T2-moderate/SAD predominant group (38.0%) in our cohort is noteworthy. Characterized by moderate inflammation and disproportionately reduced FEF_25-75_, this phenotype suggests that SAD may be a relevant feature in a subset of treatment-naive adults with asthma. Although SAD is often associated with severe asthma, our data imply its presence in early-stage uncontrolled disease. If validated, early recognition of this treatable trait could be clinically relevant, as SAD has been linked to poor asthma control.^17^^,^^18^^,^^38^ However, further research using more specific modalities, such as impulse oscillometry, is needed to confirm these observations.

For this phenotype, therapies targeting the small airways might be a primary consideration. The addition of a LAMA to ICS/LABA is supported by robust evidence to improve outcomes, partly through enhanced small airway bronchodilation,^39^ and may offer additional antitussive benefits.^40^^,^^41^ The observation by Fujiki et al^42^ that single-inhaler triple therapy provides faster relief of nocturnal symptoms in adults with treatment-naive asthma is particularly relevant for this subgroup. Although prospective validation is essential, these findings provide a rationale for considering triple therapy earlier in selected patients with evidence of SAD.

### Discordance between symptoms and inflammation

A key finding of this study was the limited correlation between symptom severity (ACQ-5) and T2 inflammatory markers. With an AUC of 0.615 for predicting T2-high status, our results suggest that symptom scores alone may not accurately reflect the underlying inflammatory burden in this specific population. Relying exclusively on symptoms could potentially lead to undertreatment of patients with high inflammation or overtreatment of patients with symptoms not driven by T2 inflammation.^43^^,^^44^ These observations support the potential value of integrating accessible biomarkers, such as Feno, into primary care assessments to complement symptom-based decision making.^45^

### Limitations and future directions

Our study has several important limitations. First, as a single-center, retrospective study with a relatively small sample size, our findings require validation in larger, multicenter cohorts. Most importantly, we did not perform an external validation of the identified clusters. Consequently, these phenotypes should be viewed as hypothesis generating rather than as definitive entities. Second, although our cohort encompassed a wide age range (18-83 years) including older adults (17.5% age ≥ 65 years), the median age was 46 years, and the majority had adult-onset asthma. Therefore, our findings—particularly the prominence of the T2-moderate/SAD predominant phenotype—may be most applicable to this demographic, and caution should be exercised when generalizing to populations with predominantly childhood-onset asthma. Third, our selection of a 3-cluster solution was driven by clinical interpretability, a recognized approach in phenotyping research,^30^ but we acknowledge that alternative analytical methods may uncover additional phenotypic distinctions. Fourth, the assessment of SAD relied primarily on FEF_25-75_, and future studies would benefit from incorporating more sensitive measures, such as impulse oscillometry.^46^ Furthermore, the inherent limitations of patient-reported outcomes, such as the ACQ-5, may have influenced our results.^47^

Finally, the ultimate goal is to translate these observational findings into clinical practice. This requires randomized controlled trials to prospectively evaluate the efficacy and cost-effectiveness of our proposed phenotype-guided therapeutic framework against standard guideline-based care. Such trials are the essential next step to confirm whether this personalized approach can truly improve long-term patient outcomes.

**Key messages**

• This study suggests that treatment-naive, uncontrolled asthma is heterogeneous, comprising 3 potential phenotypes, including a prevalent subgroup characterized by SAD.
• In this cohort, symptom severity showed limited correlation with underlying T2 inflammation, suggesting that symptom assessment alone may not fully capture the inflammatory burden.
• Incorporating objective biomarkers (eg, Feno) and functional assessments into initial evaluation may provide a more comprehensive basis for personalizing therapy compared with reliance on symptoms alone.

### Conclusion

In this retrospective analysis of treatment-naive adults with uncontrolled asthma, we identified 3 distinct phenotypes characterized by divergent inflammatory and functional profiles. Our observations suggest that symptom severity alone may not reliably predict underlying T2 inflammation and underscore the potential clinical relevance of small airway dysfunction in this population. Although these findings are hypothesis generating, they provide a conceptual framework for personalizing initial therapy through the integration of objective biomarkers and functional assessments. Prospective validation in larger, multicenter cohorts is essential to confirm the clinical utility of this approach.

### Data availability

Deidentified datasets are available on reasonable request from the corresponding author, subject to institutional review board approval and a data-sharing agreement.

## Disclosure statement

This research did not receive any specific grants from any funding agency in the public, commercial, or not-for-profit sectors.

Disclosure of potential conflict of interest: Y. Nakano received honoraria from AstraZeneca K.K. and GlaxoSmithKline. The rest of the authors declare that they have no relevant conflicts of interest.

## Figures and Tables

**Supplementary Fig E1:**